# Building Blocks to Design Liposomal Delivery Systems

**DOI:** 10.3390/ijms21249559

**Published:** 2020-12-15

**Authors:** Katarzyna Juszkiewicz, Aleksander F. Sikorski, Aleksander Czogalla

**Affiliations:** 1 Department of Cytobiochemistry, Faculty of Biotechnology, University of Wroclaw, 50-383 Wroclaw, Poland; katarzyna.juszkiewicz@uwr.edu.pl; 2 Research and Development Center, Regional Specialist Hospital, Kamieńskiego 73a, 51-124 Wroclaw, Poland; aleksander.sikorski@wssk.wroc.pl

**Keywords:** liposomes, lipid composition, drug delivery, drug release, liposomal systems, cationic lipids, cholesterol, polyethylene glycol, targeting

## Abstract

The flexibility of liposomal carriers does not just simply rely on their capability to encapsulate various types of therapeutic substances, but also on the large array of components used for designing liposome-based nanoformulations. Each of their components plays a very specific role in the formulation and can be easily replaced whenever a different therapeutic effect is desired. It is tempting to describe this by an analogy to Lego blocks, since a whole set of structures, differing in their features, can be designed using a certain pool of blocks. In this review, we focus on different design strategies, where a broad variety of liposomal components facilitates the attainment of straightforward control over targeting and drug release, which leads to the design of the most promising systems for drug delivery. The key aspects of this block-based architecture became evident after its implementation in our recent works on liposomal carriers of antisense oligonucleotides and statins, which are described in the last chapter of this review.

## 1. Introduction

**Figure 1:**
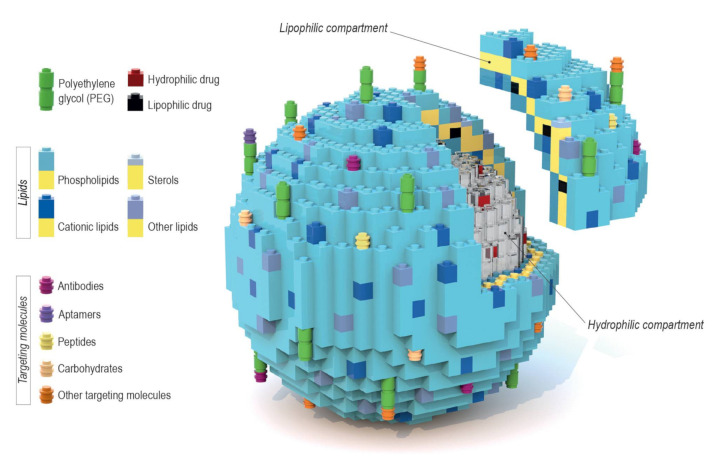
Schematic representation of a variety of liposome drug carriers’ composition, where each component is depicted as a Lego block. The liposomal envelope consists of a lipid bilayer that creates an environment for solubilizing lipophilic drugs. Inside of the liposome is an aquatic core where potential hydrophilic drugs can be entrapped. The block-like nature of this model allows for the easy engrafting of targeting particles (either directly or via polyethylene glycol (PEG)) to the liposomal surface. The modular nature of liposomal components is particularly clear in the case of formulations described in Section 5.

Liposomes are a broadly studied and constantly refined drug delivery system that provides great bioavailability, high biocompatibility, prolonged circulation time, and various targeting possibilities. What makes them such a flexible platform is their variety in terms of the encapsulated contents. The amphiphilic nature of liposomes allows them to be carriers for a broad array of therapeutic substances. In principle, hydrophilic compounds are entrapped inside the aqueous core while lipophilic ones are dissolved within the lipid bilayer, as seen in Figure 1 [1,2]. Liposomes may consist of either one lipid bilayer (unilamellar liposomes) or more (multilamellar liposomes) [3]. However, despite multiple advantages, this system may also face such problems as reduced stability which stems from chemical alterations of liposome shell components, and/or the encapsulated content, during prolonged storage (regarded as “shelf life”). In addition, liposomes tend to aggregate and undergo further undesirable changes that occur during patient treatment, e.g., after intravenous administration, which negatively impacts the pharmacodynamics and pharmacokinetics of liposomal-based products. That is why it is important to adjust different aspects of the liposomal formulation, starting with the lipid composition, to minimize possible drawbacks of the final formulation. Intravenous liposomal formulations that are intended for clinical use should possess some basic properties, such as small particle size distribution (low polydispersity index) and the long-term stability of these parameters [4]. The size distribution of liposome particles can influence stability (formulations with a lower polydispersity index value tend to show improved shelf life), cellular uptake, biodistribution, encapsulation efficiency of the drug and its release profile. The control of size distribution, as well as the lamellarity of liposome particles, is possible due to the preparation techniques such as extrusion, sonication, homogenization and freeze-thawing cycles [5]. The aforementioned techniques allow multilamellar vesicles (MLV), with a diameter of 500 to 5000 nm, to be turned into either small unilamellar vesicles (SUV) (20–100 nm) or large unilamellar vesicles (LUV) (100–1000 nm) [6]. The size distribution of liposomes is measured using dynamic light scattering, while their lamellarity can be easily estimated using transmission electron microscopy [7].

Other lipid particles used for drug delivery include first-generation solid lipid nanoparticles (SLN) and second-generation nanostructured lipid carriers (NLC). Unlike liposomes, these nanoparticles consist only of a lipidic core that can either be solid (SLN) or contain lipids in both the liquid and solid phase (NLC). The second generation of lipid nanoparticles was designed in order to overcome some of the limitations that SLN showed, such as a tendency to gelation and low drug loading capacity [8]. Although there is hardly any direct comparison of the drug encapsulation and release efficiency between liposomal systems and NLC/SLN, they seem to have similar stability, as tested after 3 months by Mennini et al. while analyzing different formulations designed for the transdermal delivery of oxaprozin. Nevertheless, in these experiments, liposomal formulations showed better permeability through both artificial lipophilic membranes and human skin than NLC dispersions [9]. There is also a third-generation commercially available drug delivery system called SmartLipids, which is characterized by a higher loading capacity of lipophilic drugs, as its matrix is composed of a chaotic structure that can include even up to ten lipid species in either the solid or liquid phase. In spite of that, it is currently dedicated only for dermal application [10]. Other formulations are composed of micelles which are built from lipophilic compartments that are surrounded by lipid monolayers, while nanoemulsions are formed by similar particles, but their molecular structure allows them to serve as a carrier for both hydrophilic and lipophilic contents. On the other hand, micelles and nanoemulsions are generally formed by various detergents, which may lead to their increased toxicity [11]. For these reasons (among others), liposomal drug delivery systems currently prevail over other lipid nanodelivery systems as they offer the greatest variety in terms of drug encapsulation efficiency and design opportunities. Currently, there are many new formulations being tested for efficient drug delivery. The design of liposomal gene delivery platforms seems to be particularly promising [12].

Liposomal systems can be characterized by three main features that influence their physicochemical properties, which have a direct impact on the pharmacokinetics and pharmacodynamics of the therapeutic compounds upon administration into the bloodstream:

- Lipid composition: The diversity and molar ratio of lipids present in the bilayer directly impact membrane fluidity, permeability, and surface charge, as well as the loading capacity of drugs.
- Drug loading and release: The nature of the encapsulated drug, which can be either hydrophilic or lipophilic. The inclusion of stimuli-sensitive lipids or other components allows for a triggered drug release under specific conditions.
- Targeting methods: Active targeting by the attachment of ligands/molecules on the vesicle surface, which are preferentially (or exclusively) recognizable by target cells/tissues, and passive targeting through usage of the enhanced permeability and retention effect (EPR) effect. The vast majority of liposomal drug formulations contain “PEGylated lipids” (lipids with attached polyethylene glycol (PEG) chains) that affect the clearance of liposomes.

We will focus solely on intravenous formulations, as they constitute a large proportion of already clinically approved drugs (see Table 1). They are the most prevalent and thus the most developed type of therapeutic liposomes [13,14]. One of the most well-known liposomal formulations available in clinical practice is Doxil, which was created to overcome the cardiotoxicity of doxorubicin and clearly shows reduced cytotoxicity when compared to the free drug. At the same time, Myocet, which is another liposomal formulation of doxorubicin, displays vastly different pharmacokinetics in comparison with Doxil, which may be partly due to the lack of PEGylated lipids in the liposomal shell. For those reasons, these two formulations are used in treatment of different types of cancer, despite encapsulating the same type of drug (see Table 1) [15].

The aim of our review is to show the interchangeability of individual liposomal components (building blocks) in light of biophysical aspects of lipid bilayers, as well as recent approaches to prepare liposomal carriers and pharmacokinetic strategies. Based on our studies on modular liposomal carriers designed for genetic drugs or statins, we provide a unique example of taking advantage of block-based architecture in fine-tuning drug delivery.

## 2. Lipid Composition

**Figure 2:**
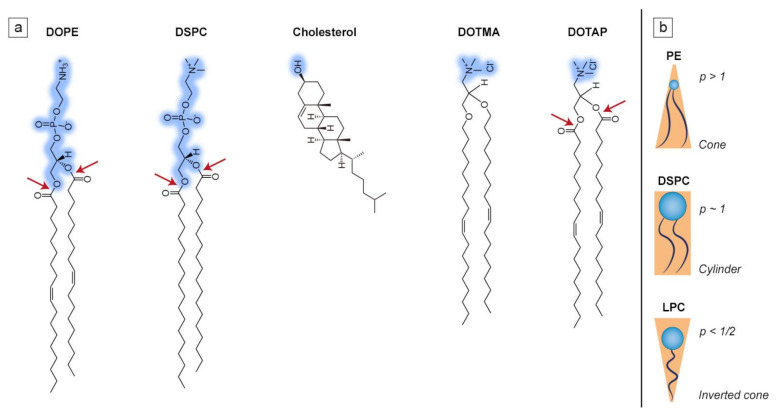
Examples of lipids commonly used in liposomal design. (**a**) Hydrophilic head groups are marked in blue while ester bonds are marked with the red arrows; (**b**) simplified representation of the influence of the packing parameter (*p* = v/al, where v is the molecular volume, a is the cross-sectional area of the head group, and l is the length of the molecule) on the molecular shape of lipids such as phosphatidylethanolamine (PE), 1,2-distearoyl-sn-glycero-3-phosphocholine (DSPC) and lysophosphatidylcholine (LPC). Figure 2b was prepared based on [32].

Lipidic amphiphiles form supramolecular aggregates in a water environment. According to the “shape concept” by Cullis and de Kruijff, the type of self-assembled structure greatly depends on the packing parameter of lipid molecules, which defines the ability to fit into a particular lipid aggregate type and is described as the polar head to hydrophobic tail ratio (see Figure 2). When the packing parameter is close to 1, lipids assemble into a bilayer which is able to form thermodynamically stable spherical vesicles known as liposomes [32]. Such is the case of the most common phospholipid found in mammalian cell membranes, 1-palmitoyl-2-oleoyl-sn-glycero-3-phosphocholine (POPC), which has a roughly cylindrical shape and is able to form liposomes on its own [33]. Most of the naturally occurring phosphatidylcholines form planar bilayers, but when mixed with conically shaped phospholipids, e.g., 1,2-dioleoyl-sn-glycero-3-phosphoethanolamine (DOPE), or any other lipids promoting bilayer curvature, liposomes are also formed, although they possess slight distortions in the bilayer structure [34]. The structures of a few selected lipids, commonly found in liposomal formulations, are presented in Figure 2a, while the correlation between molecular shape and the packing parameter is shown in Figure 2b.

The mechanical properties of the lipid bilayer depend greatly on the choice of its components. It can either be rigid and impermeable to molecules or possess a chaotic hydrocarbon tail structure which makes it more flexible [35]. The incorporation of lipids characterized by high main phase transition temperature (Tm), such as fully saturated DSPC, makes the liposomal formulation more stable and less prone to leakage of any encapsulated contents. This is because during the phase transition of the lipid bilayer the organization of the lipids changes from the ordered gel phase to a less ordered liquid crystalline phase, making it easier for some molecules to pass through the membrane [36,37]. The permeation rate of the lipid bilayer shows a peak during Tm and descends with an increasing temperature, although it is still higher than before reaching the melting point of the lipid bilayer [38]. If a specific Tm is required (e.g., while designing temperature-sensitive liposomes), replacing phospholipids with acyl chains of different types would help achieve this goal [39]. In particular, cis-unsaturated acyl chains take up more steric space, thus disrupting the packing of adjacent chains, assuring their additional flexibility. This leads to the decrease in the Tm of the entire lipid bilayer [40].

### 2.1. Phospholipids

In liposomal formulations, the majority of the lipid composition is taken up by phospholipids that can either come from natural sources or can be artificially synthetized. Natural lipids are most often obtained from soybeans, egg yolk, or bovine tissues. They are usually cheaper than their synthetic counterparts, but they are also much less homogeneous in terms of both phospholipid/impurities content and acyl chain composition. Furthermore, lipids purified from animal tissue bear a risk of contamination, which would be impermissible regarding therapeutic delivery systems, as they have to be sterile. Synthetic lipids are much more homogeneous and their production process is reproducible, meaning that each batch has exactly the same phospholipid and fatty acid composition [41]. Liposomes composed of phospholipids such as DSPC which have long, saturated fatty acid chains demonstrate higher stability when compared to other lipids containing unsaturated fatty acid chains [42]. Phosphatidylcholines are the most widely used lipids in liposomal design as they are the major component of cellular membranes, which attests to their great biocompatibility. They possess zwitterionic (at physiological pH) head groups. It was shown, however, that liposomes made of DSPC and a PEGylated lipid, which were expected to have a near-neutral zeta potential (the value of the electrokinetic potential at the slipping plane measured in colloidal dispersions), possess a slightly negative zeta potential [43]. This is also the case in liposomes made of other phosphatidylcholines such as POPC [44]. Sphingomyelins are also abundantly represented in cell membranes. They form a network of hydrogen bonds with adjacent molecules, which leads to much tighter packing in comparison to other phospholipids of comparable acyl chain composition [45]. However, there is far less structural information (including Tm) available for individual sphingomyelins. For example, the Tm for egg sphingomyelin is around 37 °C, which may result in lateral separation and increased permeability of a bilayer, composed mostly of such lipids, in physiological conditions [46,47]. This may lead to unwanted leakage of drugs encapsulated either in the aqueous core or in the lipid bilayer. Phosphatidylcholines offer more flexibility due to a variety of melting temperatures ranging from −60 to 80 °C. Generally, lipids with trans-unsaturated tails present higher Tm than phospholipids with cis-unsaturated chains commonly occurring in nature. Thus, this is yet another aspect affecting the stability and permeability of liposomes [48]. The type of phospholipid also directly impacts the encapsulation efficiency of hydrophobic drugs, as they provide a lipophilic environment that allows for solubilization of hydrophobic contents [49]. For instance, it was proven that phospholipids with shorter acyl chains offer better encapsulation efficiency of hydrophilic drugs such as suramin. The entrapment efficiency of suramin in liposomes made of 1,2-dipalmitoyl-sn-glycero-3-phosphocholine (DPPC) was higher than in liposomes containing DSPC [50]. On the other hand, formulations that included cholesterol showed a different pattern, as those consisting of DSPC (18 carbons in the acyl chain) displayed better stability and decreased leakage of the hydrophilic drug inulin than liposomes composed of either DPPC (16 carbons) or 1,2-dimyristoyl-sn-glycero-3-phosphocholine (DMPC) (14 carbons) [51].

### 2.2. Cholesterol

Most clinically approved liposomal nanomedicines (such as Doxil) contain cholesterol due to its unique properties to influence the behavior of other lipids included in the composition [52]. Cholesterol has a different impact on the behavior of the lipid membrane depending on the structure of present phospholipids. It shows higher affinity (and thus greater tendency to condense and form ordered bilayers) towards saturated phospholipids (e.g., DSPC) than to unsaturated ones, such as 1,2-dioleoyl-sn-glycero-3-phosphocholine (DOPC) [53]. However, as proven by Scherfeld et al., there is no noticeable difference regarding interactions of cholesterol with DPPC and DSPC, as these lipids mainly differ in the length of the chains by two carbon atoms. Moreover, within cellular membranes, in the presence of phospho- or sphingolipids with long, saturated chains, it forms ordered structures known as lipid rafts, which closely resemble liquid-ordered (Lo) domains observed in model membrane systems [54]. Depending on the temperature and cholesterol content, formation and coexistence of liquid-ordered, liquid-disordered (Ld) and a gel phase may occur within a bilayer. In Lo phase, lipid acyl chains indicate high order, as in the gel phase, while still retaining high rotational mobility similar to the Ld phase [55]. Thus, in living cells, cholesterol can interact with either sphingomyelins or glycerophospholipids in order to create protein–lipid assemblies of rafts, which, along with trans-membrane proteins, can take part in signal transduction [56,57]. It is generally presumed that cholesterol has a “fluidizing” effect on gel-state bilayers. It is well known that, depending on the lipid mixture, the presence of cholesterol can blur, or even eliminate the Tm of the bilayer. Such is the case of DPPC/cholesterol mixtures where the inclusion of cholesterol to approx. 30 mol % seems to abolish main phase transition of the lipid bilayer [58,59]. On the other hand, a study on DMPC/cholesterol mixtures demonstrated that cholesterol at a concentration of 66 mol % or higher may form crystals within the bilayer [60]. Yet another study, conducted in physiological conditions on DPSC and POPC bilayers, revealed that the increase in cholesterol concentration leads to the decrease in both the charge density and surface potential of lipid membranes [61]. Cholesterol’s presence may also affect the encapsulation efficiency of drugs and their release profile. It hinders the release of hydrophobic drugs such as quinine and reduces their encapsulation efficiency, as it also localizes next to long lipid chains, which creates steric hindrance. In the case of hydrophilic drugs, which interact with the polar heads of lipids (e.g., atenolol), cholesterol also limits their encapsulated amount, but it also speeds up their release rate from the liposomes [62]. Cholesterol reduces the permeability of the bilayer to water-soluble molecules since it forms strong hydrogen bonds with adjacent phospholipids and increases the overall condensation of the lipids [63,64,65]. In some cases, cholesterol appeared to be indispensable in a liposomal formulation. In regard to the research done by Matusewicz et al. with simvastatin-loaded liposomes, formulations without cholesterol led to a significant decrease in viability of the treated cells. This was probably due to a liposome-induced extraction of cholesterol from cellular membranes, which in vitro led to a lethal effect [66].

### 2.3. Charged Lipids

Depending on the lipid composition, liposomes can possess either a positive or a negative collective charge. The surface charge is one of the factors that determine the uptake of the liposomes by the reticuloendothelial system (RES) and determine how strongly the liposomes interact with serum proteins [67]. Most plasma proteins, such as albumin, possess a negative net charge at physiological pH, so they display a greater affinity to positively charged liposomes than those with a neutral charge. Some biologically active proteins will still bind to negatively charged liposomes, but to a lesser extent [68]. This is one of the reasons why anionic lipids are often present in some of the clinically approved formulations, as shown in Table 1 [69]. Phosphatidylglycerols such as 1,2-dimyristoyl-sn-glycero-3-phosphoglycerol (DMPG) seem to prevail over other negatively charged lipids (e.g., 1,2-dimyristoyl-sn-glycero-3-phospho-L-serine (DMPS)) because their presence seems to decrease the intensity of aggregation of the liposomal particles during storage when compared to other anionic lipids [70]. The exact type of used phosphatidylglycerol, whether it is 1,2-dipalmitoyl-sn-glycero-3-phosphorylglycerol (DPPG) with 16 carbon molecules in the acyl chain or DMPG with 14 carbons, is entirely dependent on the encapsulated contents and desired therapeutic effect [71].

Liposomes composed of cationic lipids are primarily used for the delivery of genetic drugs. Those synthetic lipids are composed of four functional domains (i.e., a hydrophilic head group, linker bond, backbone domain and a hydrophobic acyl chain, in analogy to phospholipids, see Figure 2a), which are carefully designed depending on the type of nucleic acid used for the delivery. A positively charged lipid is needed for efficient condensation of nucleic acids and its association with liposomes [72]. Cationic lipids not only form complexes with DNA or RNA, but also aid interaction with the negatively charged surface of cells, thus improving the transfection efficiency [73]. The differences in the structure of cationic lipids (such as acyl chain length and type of hydrophilic group) strongly affect their transfection efficiency and toxicity [74]. For instance, the ester bond of 1,2-dioleoyl-3-trimethylammonium-propane (DOTAP) is easily metabolizable, hence its toxicity is lower than other cationic lipids, such as 2-di-O-octadecenyl-3-trimethylammonium propane (DOTMA) (see Figure 2a) [75]. Most of the cationic lipids used in gene delivery are characterized by double hydrophobic chains, as this allows them (to a small degree) to induce hexagonal phases after cellular internalization, which leads to increased transfection efficiency. Single-tailed lipids behave like surfactants and form micelles, which greatly increases their cytotoxicity [76]. On the other hand, cationic lipids derived from cholesterol are protein kinase C (PKC) inhibitors, which increases their toxicity. A similar issue concerns the polar group of the cationic lipids, as quaternary ammonium amphiphiles are stronger PKC inhibitors than tertiary ones. This is why counterparts possessing a heterocyclic ring instead of an amine head group are significantly less toxic [77]. Another reason why cationic liposomes are one of the best choices for gene delivery is their protective abilities. DNA/RNA is fully protected only within net positive complexes, as this stands for a totally condensed nucleic acid that is inaccessible to potential endonuclease activity. Complexes of net negative charge do not protect DNA/RNA [78].

Cationic liposomes, which are known to be efficient carriers of nucleic acids, are not exclusively composed of cationic lipids. The presence of so-called helper lipid is usually required, and they fine tune the formulation behavior and its efficiency. For example, addition of DOPE to the composition greatly raises the transfection efficiency when compared to other lipids such as DOPC. This is because DOPE-containing bilayers undergo a transition in the acidic environment (pH around 5.0) that is found in late endosomes to hexagonal phases which facilitate endosomal escape of the nucleic acid [79]. In general, the addition of neutral lipids with a small head group and bulky acyl chain (characterized by packing parameter in the range of 0.5–1) increases membrane fusion [33]. Importantly, the addition of neutral lipids to the formulation also decreases the toxicity of lipoplexes [80]. Generally, a less stable liposomal membrane is desired, because it promotes the release of nucleic acids inside the targeted cells. However, liposomes including membrane-stabilizing cholesterol (at a molar ratio of 1:1 to the cationic lipid DOTMA) proved to be more efficient for transfection in vivo than those including DOPE (at a molar ratio of 1:1 to DOTMA). This is due to the fact that cholesterol increases the packing density of phospholipid molecules, making it more difficult for serum proteins to adsorb to the liposomal surface [81]. On the other hand, in vitro experiments lead to a different conclusion, as DOPE-including liposomes achieved higher transfection levels than those including cholesterol. It is not uncommon that while some liposomal formulations show great transfection efficiency in initial tests conducted on cell lines, they do not perform as well in later in vivo experiments [82]. This happens due to the differences in pharmacokinetics which mostly stem from the presence of other cells and systems (such as RES) in vivo. In the case of cationic liposomes, the release of liposomal cargo is achievable due to the presence of positively charged head groups of the cationic lipids. The same mechanism occurs with polyethylenimine (PEI)/DNA polyplex nanoparticles. After entry to the cell, the lipoplexes or lipopolyplexes can escape the endosome through the “proton sponge effect”, thus preventing unwanted degradation of the encapsulated genetic material [83]. This mechanism consists of a protonation of the amino groups of PEI or cationic lipids, which leads to a high osmotic pressure inside the endosome. This stimulates water entrance and swelling that eventually leads to late endosome bursting and release of liposomal contents into the cytosol [84]. Polyplexes, such as PEI/DNA, are sometimes encapsulated within liposomes for efficient gene delivery. These structures are known as lipopolyplexes and, while they will not be described in this paper, they have been reviewed in detail by Rezaee et al. [85].

Liposomes with a notable surface charge display decrease circulation time, in comparison to neutral liposomes, as they are quickly opsonized. Nevertheless, the type of charge is not inconsequential, as it dictates which kind of plasma proteins are adsorbed to the liposomal surface and is responsible for the further fate of opsonized particles [86]. Proteins with isoelectric points lower than 5.5 (such as albumin) preferentially bind to the positively charged particles, while proteins with an isoelectric point greater than 5.5 (e.g., immunoglobulin G (IgG)) mostly bind to negatively charged particles. The negative net charge of liposomes can be used as a liver targeting factor since binding of IgG leads to hepatic uptake by Kupffer cells [87]. Furthermore, Campos-Martorell et al. conducted a series of experiments on liposomal formulations of simvastatin that possessed neutral, negative and positive charges. The results show that while both neutral and negatively charged liposomes were detectable in brain and plasma, the positively charged ones could not cross the blood–brain barrier (BBB). Because it is possible for negatively charged liposomes to cross the BBB, they can be used for both liver- and brain-targeted drug delivery [88].

## 3. Drug Loading and Release

There are many ways to encapsulate drugs into liposomes, but all these methods generally fall under one of two categories. Passive loading is carried out during the formation of the liposomes where either the dry lipid film is formed in the presence of a hydrophobic drug or the lipid film is rehydrated with the use of a hydrophilic drug solution. Unfortunately, the encapsulation efficacy of hydrophilic drugs is usually low. This method can also cause a rapid, uncontrolled release of entrapped contents from the liposomes [89]. Active loading often depends on either an ion or a pH gradient across the membrane of already preformed liposomes. An in depth discussion about these methods can be found in the review by Gubernator [90]. The properties of an encapsulated drug make a major difference in their liposome-modulated bioavailability. For example, the release rate of the hydrophobic drug dibucaine is much lower than that of the hydrophilic 5-fluorouracil when encapsulated within multilamellar liposomes formed with egg phosphatidylcholine and cholesterol. This difference was further increased in the case of negatively charged liposomes where the hydrophobic drug release was inhibited due to the charge on the liposomal surface [91]. It was also demonstrated on hydrophobic dexamethasone that the longer the acyl chains of lipids forming the vesicles are, the smaller the space between those acyl chains in a bilayer is, and, in consequence, there is less space for a hydrophobic drug to be incorporated, which reduces its encapsulation efficiency [92]. It is possible to achieve tighter control over the release of the liposomal cargo through the use of stimulus-responsive liposomes, which become metastable under certain conditions, such as pH, redox potential or temperature [93]. These aspects are unique to a disease condition and pathological state of tissues, because inflammation is always accompanied by local hyperthermia. For the design of thermosensitive liposomes lipids such as 1-myristoyl-2-palmitoyl-sn-glycero-3-phosphocholine (MPPC) (Tm = 35 °C) and 1-myristoyl-2-stearoyl-sn-glycero-3-phosphocholine (MSPC) (Tm = 40 °C) are commonly used [94]. The Tm of these lipids is around the physiological temperature, rendering the lipid bilayer more permeable during the phase transition temperature, which occurs in the tissue in which the inflammation takes place [95]. Local hyperthermia can also be artificially induced by near-infrared (NIR) radiation that is able to penetrate into deep tissues. This was the case with thermosensitive liposomes carrying both the NIR-absorbing dye indocyanine green and the anticancer drug doxorubicin. This synergic solution allowed for the effective release of encapsulated contents into the cancer cells [96]. Nguyen et al. proposed a similar concept, but instead of the dye they used gold nanorods (entrapped inside the liposomal aqueous core alongside doxorubicin) that have high absorbance of NIR light, which allowed for liposomes to be easily heated up, releasing the encapsulated doxorubicin. These liposomes also took advantage of both passive and active targeting, as they had folate grafted to their surface via PEG chains and their particle size was smaller than 150 nm [97] (see the next section). Yet another way of facilitating drug release from thermosensitive liposomes is with the use of radiofrequency ablation (RFA). This procedure involves high-frequency electrical pulses which pass through an electrode, creating a small region of heat in a selected area. A phase III clinical study was conducted on a combination of lyso-thermosensitive liposomal doxorubicin (ThermoDox) with RFA. ThermoDox contains a lysolipid monostearoyl-phosphatidylcholine (MoSPC) that forms defects above its Tm (40 °C) that aid the release of encapsulated contents [98]. An animal study was conducted in order to optimize heating time and then this combinational therapy was tested on patients with hepatocellular carcinoma, and was found to significantly improve their overall survival [99,100,101]. Chen et al. developed a thermoresponsive liposomal system for extracellular delivery of doxorubicin. Its key component, ammonium bicarbonate, which is used in generating a transmembrane gradient for the encapsulation of the drug, decomposes to carbon dioxide bubbles upon heating. This process generates defects in the lipid bilayer, leading to the quick release of the encapsulated doxorubicin. These liposomes are also more stable in blood plasma and have a longer circulation time when compared to lysolipid liposomes, such as ThermoDox [102,103]. With the aim of improving the utility of ABC liposomes for potential chemotherapy, Cheng et al. modified this formulation for intracellular drug delivery. They used PEGylated 1,2-distearoyl-sn-glycero-3-phosphorylethanolamine (DSPE-PEG) with attached folate. Such modified liposomes effectively released doxorubicin in both low pH of 6.4 (which is present in tumors) and mild hyperthermia (42 °C), while maintaining intravenous stability at normal body temperature (around 37 °C) [104]. In solid tumors, the intratumoral pH value is slightly lower than the pH of blood and surrounding tissues, which is taken into consideration when composing pH-sensitive liposomal formulations. Those liposomes enter the tumor tissue and quickly become destabilized while releasing the encapsulated contents. However, pH values differ in endosomes and in the tumoral environment. All these elements must be taken into account when designing a pH-sensitive liposomal formulation and choosing lipids with the desired Tm [105,106]. DOPE is the most popular choice as thanks to its cone shape it forms hexagonal phases. However, due to this, DOPE cannot form lipid bilayers by itself in neutral pH and requires the presence of weakly acidic amphiphilic lipids such as cholesteryl hemisuccinate or cylindrically shaped lipids such as PC [73,105,107]. Nonetheless, the fusogenic properties of DOPE seem to be inhibited in the presence of serum, as opsonins interact with the DOPE-containing bilayer. Redox potential can also be used as a stimulus, as in the case of liposomes designed for the treatment of human osteosarcoma. The surface of liposomes was coated with chitooligosaccharides via disulfide bonds. The intracellular environment (especially in cancer tissues) is much more reductive than the extracellular environment. This means that liposomes did not show any unwanted drug leakage in physiological conditions but were destabilized by reducing agents such as dithiothreitol or glutathione [108].

## 4. Targeting and Clearance

**Figure 3:**
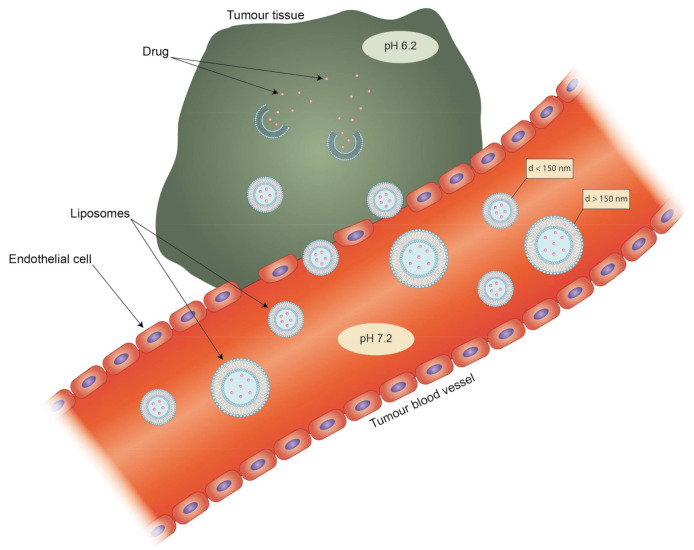
The complementary effect of enhanced permeability and retention effect (EPR) and pH sensitivity of liposomes. Only liposomes with a diameter (d) smaller than 150 nm are able to pass through leaky endothelium cells in blood capillaries into tumor tissue. Blind-ended tumor blood vessels lack lymphatic drainage, thus increasing the accumulation of liposomes.

Liposomes can be the subject of active and passive targeting. The latter depends on a phenomenon called the enhanced permeability and retention effect (EPR) in the environment of tumors. Rapid tumor vascularization leads to the formation of immature tumor vessels inside the tumoral mass characterized by high permeability, which leads to the accumulation of nanoparticles smaller than 150 nm which are able to cross these vessels. Moreover, due to the abrupt ending of these vessels, there is a lack of functional lymphatic drainage, so the clearance of any accumulated particles is hindered (Figure 3) [109]. The EPR effect does not occur only in tumors, as inflamed tissues are also characterized by enhanced vascular permeability, as for example in the case of rheumatoid arthritis. Jia et al. tested a liposomal formulation of the hydrophobic drug dexamethasone on an adjuvant-induced arthritis (AIA) rat model. While the free drug showed a decrease in inflammation, it also led rats to develop hyperglycemia. The liposomes seemed not to have such a strong side effect and also showed better accumulation in inflamed tissues [110]. Targeting via size is also effective when the target is a part of the RES. Particles in the range of 100 nm to 150 nm are preferentially taken up by phagocytes and accumulate in the liver [111]. Liposomes that extend beyond 150 nm are characterized by rapid uptake by the mononuclear phagocytic system (MPS), which matters in the treatment of such diseases as leukemia and rheumatoid arthritis [112]. However, passive targeting also has its drawbacks. The transporters that take part in the clearance of drugs and other molecules from cells are usually overexpressed in the case of cancer cells. This may lead to the development of multiple-drug resistance of the target cells and render the drug treatment ineffective [113]. This could be counteracted with a targeted liposomal formulation possessing an aptamer-labelled surface. Such liposomes were designed by Powell et al. for selective delivery of siRNA silencing the P-gp transporter gene found in metastatic breast cancer cells [114].

**Figure 4:**
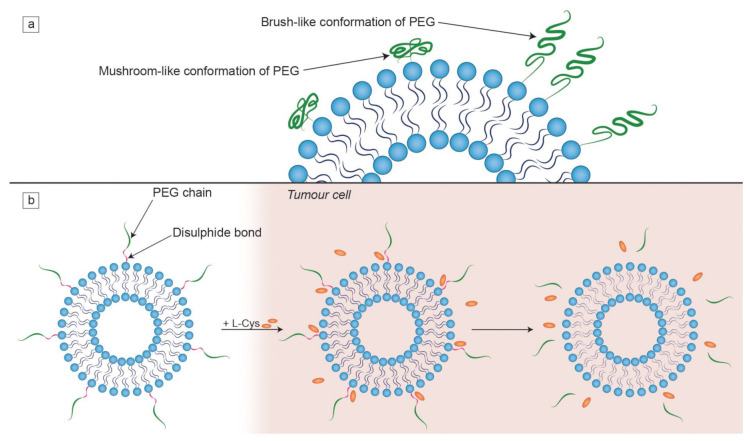
Characteristics of PEG chains on the liposomal surface. (**a**) The dependence of the PEG chain conformation on its surface density. When PEG chains are engrafted at a higher density, the brush-like conformation is favoured; (**b**) the mechanism of detachment of PEG chains from a liposome by the L-cysteine (L-Cys) that is found at an elevated concentration in tumor cells. This illustration was developed based on a procedure described in a study by Kuai et al. [122].

Effective targeting is one of the major aims in designing an efficient liposomal formulation for drug delivery. Another important element to take into consideration is the level of clearance of liposomes. In order to reduce the MPS uptake, several physico-chemical properties of the bilayer that forms liposomes can be modified [39]. Denser packing of lipids in the bilayer means reduced absorption of opsonins, which can be achieved by the incorporation of cholesterol, as mentioned before. It must be noted that while smaller liposomes evade RES more easily, their aqueous internal compartment has a smaller volume, meaning less available space for hydrophilic drugs [3]. The blood circulation time of the liposomes decreases with increasing size of the particles and net charge density [115]. The rigidity of the membrane can be increased by incorporating lipids with high Tm, such as DSPC (Tm = 55 °C), which results in the reduction in the MPS uptake [107]. The most prevalent way of decreasing the uptake by the RES is grafting PEG chains to the liposomal surface. This happens due to the fact that PEG establishes a steric barrier on the liposomal surface, which reduces opsonization by the serum components in vivo, thus positively influencing pharmacokinetics of liposomes [39,116]. The conformation of PEG chains depends on their grafting density. At higher densities, PEG is found in a brush-like conformation, while mushroom-like PEGs dominate in lower densities (Figure 4a). These conformations yield different degrees of hydrophobic shielding, as this effect is greater in the case of brush-like PEG chains. Longer PEG chains provide better protection against plasma proteins than shorter ones [117,118]. For instance, Doxil consists of “stealth” liposomes, because PEGylation reduces their RES uptake, greatly increasing their circulation time [119]. On the other hand, repeated injections of PEGylated formulations may lead to the production of anti-PEG antibodies that absorb to the liposomal surface [120]. At the same time, some alternatives to PEG are also being tested so as to combat the problem of PEG immunogenicity. Some hydrophilic polymers (such as polyglycerols and polyoxazolines) and zwitterionic polymers have shown a comparable or improved results in producing stealth formulations [121].

Nevertheless, the binding of opsonins to therapeutic nanoparticles can also work in favor of targeted delivery. Such is the case of the formulation designed by Zhang et al. for treatment of hereditary transthyretin-mediated (hATTR) amyloidosis. After administration into the bloodstream, liposomes (called “lipid nanoparticles”) are opsonized by apolipoprotein E and taken to the liver, where they bind to the surface of hepatocytes due to the presence of apolipoprotein E receptors. This phenomenon allows them to effectively deliver encapsulated siRNA into hepatocytes and silence a mutated version of the TTR gene, which was tested on patients suffering from hATTR amyloidosis [123].

Active targeting consists of conjugating various types of ligands to the liposomal surfaces such as antibodies, sugars, lectins and proteins, as reviewed by Toporkiewicz et al. [124]. Targeting agents may be bound directly to lipid anchors on the liposomal bilayer or attached by a linker such as PEG (Figure 1). The second option is preferred because adjacent PEG chains, which are included in the formulation for the RES evasion, may sterically inhibit the binding of ligands (if found closer on the liposome surface) to the target cells [125]. This is especially significant to antibodies, as those directly attached to the liposomal bilayer surface have their antigen binding abilities partly inhibited by PEG chains of a molecular weight of 5000 Da [126]. Another set of experiments showed that PEG molecules of different chain lengths show a different degree of inhibition of plasma immunoprotein binding. PEG(600) inhibited the C1q protein complex (a part of the innate immune system, called complement) binding to cardiolipin containing liposomes by approx. 50%, while the presence of PEG(1000) and PEG(2000) resulted in total binding inhibition [127]. Moreover, PEG chains on the liposomal surface can prevent endosomal escape of a drug. It may both suppress electrostatic interactions required for effective cellular uptake and interfere with the fusion of the endosomal membranes with those of liposomes [128]. Thus, in order to overcome these limitations, a biochemical approach to detaching PEG from the lipid anchor under specific conditions was developed [93,129]. It happens by means of attachment of PEG chains to lipid anchors via a disulfide bond that is easily cleavable by exogenous L-Cys found in tumor tissue (Figure 4b) [122]. There are many other potential liposomal components sensitive to various stimuli—for instance, hydrazone bonds break in an acidic environment, which is characteristic for pathological tissues [130]. Attaching ligands without using PEG is possible using other types of linkages containing various groups binding, e.g., tagged recombinant proteins. For example, synthetic lipid 1,2-dioleoyl-sn-glycero-3-[(N-(5-amino-1-carboxypentyl)iminodiacetic acid)succinyl] (DGS-NTA(Ni)) binds to polyhistidine tags. This was used in an experiment where a His-tagged p24 was bound to nanovesicles with surface-chelated nickel. The effectiveness of this liposomal formulation was confirmed both in vitro and in vivo in mouse models [131].

The liposomal bilayer provides a flexible platform for possible targeting. The ability of attaching multiple surface ligands allows for even more specific targeting [132]. It has been shown than both PEG chains and antibodies can be attached to form “stealth” immunoliposomes that both bind to the targeted ligands and evade RES uptake [133]. Nonetheless, such liposomes show reduced cellular uptake in comparison to non-PEGylated immunoliposomes. Some formulations take advantage of both active targeting and combined therapy. Lv et al. designed and tested a formulation consisting of lysolipid-containing thermosensitive liposomes. They contained both marimastat and paclitaxel, and had hyaluronic acid grafted on the surface, thus showing a strong affinity for CD44 receptors, which are overexpressed in cancer tissues. Marimastat is a strong inhibitor of metastasis, but it is not sufficient to eliminate cancer cells, so an effective cytostatic drug, paclitaxel, was also included. Those liposomes crossed into the tumor microenvironment successfully, releasing the encapsulated contents due to the local hyperthermia [134]. On the other hand, Lakkadwala and Singh grafted transferrin for targeting to the liposomal surface but also used the cell-penetrating protein FVYLI (to increase cellular uptake) attached by the PEG chain to DSPE. These dual surface-functionalized liposomes were supposed to serve as a treatment for glioma, as they are able to cross the BBB. The successful drug release of both doxorubicin and erlotinib was tested on glioblastoma (U87) cells that served as an in vitro brain tumor model [135]. This is a large step towards the preparation of multifunctional nanocarriers for cancer therapy.

## 5. Individual Blocks That Make the Difference

**Figure 5:**
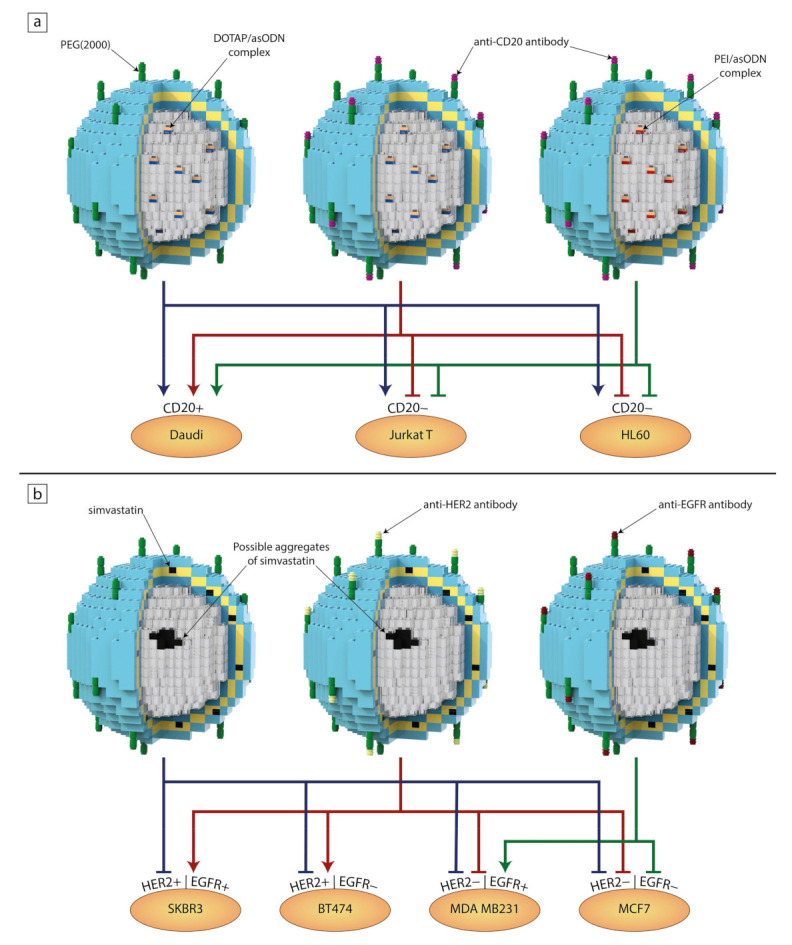
The interchangeability of individual blocks resulting in different or synergistic effects on individual cell lines after treatment with liposomal formulations containing antisense oligonucleotide (asODN) complexes (**a**) [136,137] and simvastatin (**b**) [66,138]. Arrows show a noticeable decrease in cell viability and/or interactions with cells, while blind-ended arrows indicate a lack of a significant effect after the addition of liposomes to the cell culture medium.

As mentioned above, one of the main reasons why liposomes are such an attractive drug delivery system is the modularity/interchangeability of their components. Usually, the design of a liposomal formulation starts with optimizing a lipid composition of the bilayer, so the chosen entrapped drug exhibits the best possible encapsulation efficiency and stability. Once that is established, one can experiment with engrafting different targeting molecules to the liposomal surface depending on the desired therapeutic effect. It is quite a simple task to replace just one or two components in order to generate a new formulation targeted towards different types of cells. This is why an analogy to Lego blocks is used in Figure 1. In a similar way, in Figure 5, we summarize the results of our recent studies discussed below.

Wyrozumska et al. designed an untargeted liposome-coated lipoplex (L-cl) containing antisense oligonucleotides (asODNs) against the *BCL-2* gene. The liposomal bilayer consisted of hydrogenated egg phosphatidylcholine (HEPC), 3b-(N-[dimethylaminoethane]carbamoyl)cholesterol) (DC-Chol), DOPE and DSPE-PEG. The negatively charged asODN molecules were complexed with a positively charged lipid, DOTAP. This formulation showed great stability in human serum as well as shelf life after 12 months. In vitro tests proved that L-cl significantly decreased Bcl-2 protein expression in Jurkat T and Daudi cells. In addition, L-cl reduced the survival rates of Jurkat T, HL60, Daudi and white blood cells isolated from patients with acute myeloid leukemia. Liposomes accumulated in the livers and spleens of NOD/SCID mice, in which Daudi Burkitt’s lymphoma was engrafted, and were detectable in the bloodstream up to 24 h after injection. This experiment shows that passive targeting alone could produce satisfactory results both in vitro and in vivo [136].

Meissner et al. also focused on liposomal lipoplexes carrying anti-*BCL-2* asODNs composed of similar lipid composition, but they attached rituximab (a therapeutic anti-CD20 antibody) to the liposomes via a maleimide PEG derivative (DSPE-PEG-Mal). Moreover, they tested two formulations with varying DNA complexing factors. One contained asODNs complexed with DOTAP (L-D) and the other had asODNs complexed with PEI (L-P). The non-specific toxicity was tested on cell lines that do not overexpress CD20 (Jurkat T, HL60, HEL) and white blood cells isolated from the peripheral blood of healthy volunteers. As expected, both targeted formulations showed toxicity only towards CD20-expressing Daudi cells, which was manifested by a reduced Bcl-2 protein level and induction of a substantial level of apoptosis. As in the research by Wyrozumska et al. [136], in vivo experiments were conducted on NOD/SCID mice with engrafted Daudi Burkitt’s lymphoma. Both L-D and L-P were detectable in the bloodstream for 24 h after injection and accumulated in the engrafted tumors to a much greater degree than their non-targeted counterparts. These liposomal formulations hindered tumor development without any obvious negative effects on the animals. It seems that the only noticeable difference between L-D and L-P is found in their positive zeta potential values, as it was 22 and 5 mV, respectively. Both systems revealed good therapeutic efficacy regardless of the type of factor used for complexing anti-*BCL-2* asODNs [137].

Another example is in the research of Matusewicz et al., who proposed immunoliposomes targeted against breast cancer cells overexpressing human epidermal growth factor receptor 2 (HER2) encapsulating a highly lipophilic drug, simvastatin. First, they carefully tested four lipid compositions in order to achieve the best stability and drug encapsulation. The final composition consisted of hydrogenated soya bean phosphatidylcholine (HSPC), DSPC, cholesterol, DSPE-PEG and DSPE-PEG-Mal. The FDA-approved humanized monoclonal antibodies (trastuzumab/Herceptin) were attached using the same procedure as described in the previously mentioned study [137]. Targeted and non-targeted liposomal formulations were tested on cell lines that overexpressed HER2 (SKBR3 and BT474) and on those that did not (MDA MB 231). As expected, neither formulation showed a decrease in the viability of cells without HER2 overexpression, while immunoliposomes induced apoptosis and inhibited the signaling pathway involving Akt and Erk in the HER2-overexpressing SKBR3 cell line [66]. In another study, Matusewicz et al. took their previously established liposomal composition and exchanged trastuzumab for an anti-EGFR therapeutic antibody (cetuximab) with the hope of treating triple-negative breast cancers, as about half of them overexpress the epidermal growth factor receptor (EGFR). Viability tests were carried out on a cell line overexpressing EGFR (MDA MB 231) as well as on MCF7 cells that had low EGFR expression. Immunoliposomes proved to be far less toxic for the MCF7 cells, while inducing apoptosis and inhibiting the Akt signaling pathway in the MDA MB 231 cell line. This time, immunoliposomes were tested on NOD/SCID mice (bearing an MDA MB 231 xenograft) during the early stages of tumor formation as well as on animals with already preformed tumors. Targeted liposomes were detectable in the bloodstream up to 24 h after injection and, after accumulating in tumors, remained there for 48 h. Regarding tumoral growth inhibition, targeted formulation showed about a 17% decrease in growth until five days after the last dose, when the tumors suddenly underwent rapid growth [138].

## 6. Conclusions

Liposomal delivery systems offer great variety regarding both gene and drug therapy, with a number of formulations already approved for clinical use (Table 1). Taking a careful approach in designing each individual component, starting with lipids forming the lipid bilayer and ending with surface engrafted particles, is essential for achieving the desired therapeutic effect. The composition does not have to be limited to lipids only, as many long-circulating liposomes incorporate PEG chains of various length. Diversity in available “building blocks” (Figure 1) enables advanced targeting towards cells, as well as triggered release of encapsulated contents under specific conditions. Exchanging just one component can result in a change in the properties of liposomal particles. However, as described in the fifth section, it is not uncommon to use an already established lipid composition for the delivery of multiple therapeutic substances. It is safe to assume that, due to their “adaptability”, liposomes are still a valid and evolving delivery system.

## Figures and Tables

**Table 1:** List of liposomal drug products for injection clinically approved by European Medicines Agency (EMA) and Food and Drug Administration (FDA).

Drug	Product Name	Route of Administration	Lipid Composition (Molar Ratio ^1^)	Treatment	Ref.
Amphotericin B	Abelcet	Intravenous	DMPC, DMPG (7:3)	Systemic fungal infections	[16]
	Ambisome	Intravenous	HSPC, DSPG, cholesterol (2:0.8:0.4)	Systemic fungal infections	[17]
Bupivacaine	Exparel	Supraperiosteal Injection	DEPC, DPPG, cholesterol, tricaprylin	Postsurgical local analgesia	[18]
	Nocita	Supraperiosteal Injection	DEPC, DPPG, cholesterol, tricaprylin	Postsurgical local analgesia (for dogs only)	[19]
Cytarabine	Depocyt	Spinal	DOPC, DPPG, cholesterol, triolein (7:1:11:1)	Lymphomatous meningitis	[20]
Daunorubicin	DaunoXome	Intravenous	DSPC, cholesterol (2:1)	Kaposi’s sarcoma	[21]
Doxorubicin	Doxil/Caelyx ^2^	Intravenous	HSPC, cholesterol, DSPE-PEG (2000) (56:39:5)	Kaposi’s sarcoma	[22]
	Lipodox	Intravenous	DSPC, cholesterol, DSPE-PEG (2000) (56:39:5)	Kaposi’s sarcoma, ovarian/breast cancer	[23]
	Myocet liposomal ^3^	Intravenous	EPC, cholesterol (55:45)	Metastatic breast cancer	[24]
Inactivated hepatitis A virus	Epaxal	Intramuscular	DOPC, DOPE (75:25)	Hepatitis A	[25]
Inactivated hemagglutinin of influenza virus strains A and B	Inflexal V	Intramuscular	DOPC, DOPE (75:25)	Influenza	[26]
Irinotecan	Onivyde	Intravenous	DSPC, MPEG-2000-DSPE	metastatic adenocarcinoma of the pancreas	[27]
Mifamurtide	Mepact ^2^	Intravenous	POPC, DOPS (7:3)	High-grade non-metastatic osteosarcoma	[28]
Morphine sulfate	DepoDur	Epidural	DOPC, DPPG, cholesterol, triolein (7:1:11:1)	Pain management	[29]
Verteporfin	Visudyne	Intravenous	DMPC, EPG (5:3)	Age-related macular degeneration, pathologic myopia, ocular histoplasmosis	[30]
Vincristine sulfate	Marqibo	Intravenous	Sphingomyelin, cholesterol (6:4)	Acute lymphoblastic leukaemia	[31]

HSPC—hydrogenated soya bean phosphatidylcholine; DSPG—1,2-distearoyl-sn-glycero-3-phosphoglycerol; DEPC—1,2-dierucoyl-sn-glycero-3-phosphocholine; DSPE-PEG(2000)—1,2-distearoyl-sn-glycero-3-phosphoethanolamine-N-[amino(polyethylene glycol)-2000]; EPC—egg phosphatidylcholine; MPEG-2000-DSPE—1,2-distearoyl-sn-glycero-3-phosphoethanolamine-N-[biotinyl(polyethylene glycol)-2000]; DOPS—1,2-dioleoyl-sn-glycero-3-phospho-L-serine; EPG—egg phosphatidylglycerol. ^1^ If available. ^2^ Outside the United States, Doxil is known as Caelyx. ^3^ These formulations are only approved by EMA and not by FDA.

## Abbreviations

MLV	multilamellar vesicles
SUV	small unilamellar vesicles
LUV	large unilamellar vesicles
SLN	solid lipid nanoparticles
NLC	nanostructured lipid carriers
EPR	enhanced permeability and retention effect
PEG	polyethylene glycol
POPC	1-palmitoyl-2-oleoyl-sn-glycero-3-phosphocholine
DOPE	1,2-dioleoyl-sn-glycero-3-phosphoethanolamine
PE	phosphatidylethanolamine
DSPC	1,2-distearoyl-sn-glycero-3-phosphocholine
LPC	lysophosphatidylcholine
Tm	main phase transition temperature
PC	phosphatidylcholine
DPPC	1,2-dipalmitoyl-sn-glycero-3-phosphocholine
DMPC	1,2-dimyristoyl-sn-glycero-3-phosphocholine
DOPC	1,2-dioleoyl-sn-glycero-3-phosphocholine
Lo	liquid-ordered
Ld	liquid-disordered
RES	reticuloendothelial system
DMPG	1,2-dimyristoyl-sn-glycero-3-phosphoglycerol
DMPS	1,2-dimyristoyl-sn-glycero-3-phospho-L-serine
DPPG	1,2-dipalmitoyl-sn-glycero-3-phosphorylglycerol
EMA	European Medicines Agency
FDA	Food and Drug Administration
HSPC	hydrogenated soya bean phosphatidylcholine
DSPG	1,2-distearoyl-sn-glycero-3-phosphoglycerol
DEPC	1,2-dierucoyl-sn-glycero-3-phosphocholine
DSPE-PEG(2000)	1,2-distearoyl-sn-glycero-3-phosphoethanolamine-N-[amino(polyethylene glycol)-2000]
EPC	acidic egg phosphatidylcholine
MPEG-2000-DSPE	1,2-distearoyl-sn-glycero-3-phosphoethanolaMine-N-[biotinyl(polyethylene glycol)-2000]
DOPC	1,2-dioleoyl-sn-glycero-3-phospho-L-serine
EPG	egg phosphatidylglycerol
DOTAP	1,2-dioleoyl-3-trimethylammonium-propane
DOTMA	2-di-O-octadecenyl-3-trimethylammonium propane
PEI	polyethylenimine
IgG	immunoglobulin G
BBB	blood–brain barrier
MPPC	1-myristoyl-2-palmitoyl-sn-glycero-3-phosphocholine
MSPC	1-myristoyl-2-stearoyl-sn-glycero-3-phosphocholine
MoSPC	monostearoyl-phosphatidylcholine
NIR	near-infrared
RFA	radiofrequency ablation
DSPE-PEG	PEGylated 1,2-distearoyl-sn-glycero-3-phosphorylethanolamine
AIA	adjuvant-induced arthritis
MPS	mononuclear phagocytic system
d	diameter
L-Cys	L-cysteine
hATTR	hereditary transthyretin-mediated
L-cl	liposome-coated lipoplex
HEPC	egg phosphatidylcholine
DC-Chol	3b-(N-[dimethylaminoethane]carbamoyl)cholesterol)
HER2	human epidermal growth factor receptor 2
HSPC	hydrogenated soya bean phosphatidylcholine
EGFR	epidermal growth factor receptor
PKC	protein kinase C
